# A Review of the Ethnomedicine, Phytochemistry, Pharmacology and Toxicological Studies on *Ptaeroxylon obliquum* (Thunb.) Radlk. (Rutaceae)

**DOI:** 10.3390/plants14121746

**Published:** 2025-06-06

**Authors:** Ntanganedzeni Makumbane, Sanah Malomile Nkadimeng, Edward Thato Khunoana, Thanyani Emelton Ramadwa

**Affiliations:** Department of Life and Consumer Sciences, College of Agriculture and Environmental Sciences, Florida Campus, University of South Africa, Private Bag X6, Florida 1710, South Africa; 17490200@mylife.unisa.ac.za (N.M.); nkadism@unisa.ac.za (S.M.N.); khunoet@unisa.ac.za (E.T.K.)

**Keywords:** *Ptaeroxylon obliquum*, phytochemistry, pharmacology, ethnomedicine, toxicology, obliquumol

## Abstract

*Ptaeroxylon obliquum* (Thunb.) Radlk. (Rutaceae) is traditionally used for a range of purposes, including ethnoveterinary medicine and to treat various human ailments such as tuberculosis, inflammatory diseases, and bacterial and fungal infections. This review aims to comprehensively summarize the traditional uses, phytochemistry, toxicology, in silico, and pharmacological activities of *P. obliquum* and discuss the advances made to date. The phytochemistry of *P. obliquum* revealed the abundance of secondary metabolites such as coumarins and chromones, essential oils, and several other classes of bioactive compounds. A total of 80 secondary metabolites have been reported from this plant species. In vitro studies on *P. obliquum* explored its therapeutic potential and reported pharmacological properties such as antifungal, antibacterial, antiparasitic, antimycobacterial, anti-inflammatory, and antiproliferative activities. This review highlights the diversity of the medicinal use of *P. obliquum* and encourages its preservation. Future research should focus on the efficacy of *P. obliquum’s* most promising bioactive compounds, and the ADME (absorption, distribution, metabolism, and excretion) pharmacological activities may help determine therapeutic potential in in vivo animal models and validate the wide range of traditional uses of *P. obliquum*.

## 1. Introduction

*Ptaeroxylon obliquum* (Thunb.) Radlk. is a plant species that belongs to the order Sapindales and the Rutaceae family. While the genus Ptaeroxylon had been understandably placed in the Ptaeroxylaceae sub-family, its taxonomy has been previously contradicted, placed under different families including Meliaceae, Sapinadaceae, and Cneoraceae [1,2]. Previous investigations were guided by morphological classification, such as the presence of secretory cells on leaves that first led to the placement of Ptaeroxylon in the Meliaceae family, of which the decision was later challenged due to distinct pollen grains and secondary xylem [3,4]. Studies on the molecular composition of *P. obliquum* have revealed some uniqueness, such as the presence of coumarins, chromones, and liminols, which are mostly shared between Ptaeroxylon and Cedrelopsi, the only two members of the Ptaeroxylaceae sub-family [2,5].

**Figure 1:**
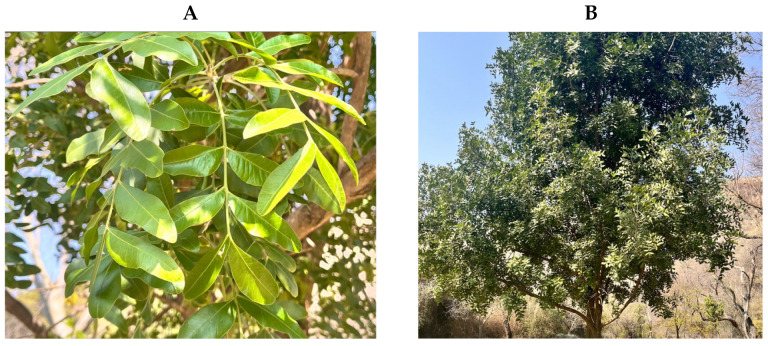
*Ptaeroxylon obliquum* leaves (**A**) and tree (**B**) taken at Walter Sisulu National Botanical Gardens at Roodepoort, Gauteng, South Africa.

*Ptaeroxylon obliquum* is commonly known as sneezewood, a name that arises from the plant’s potent irritation that has influenced its use by the Xhosa people as snuff [6]. The plant is identified by its smooth pale gray bark of the young stem that ages to a fissured blotched bark, leaves of three to eight pairs of dark green opposing asymmetrical leaflets (Figure 1), and small sweetly scented white, orange-centered flowers with four petals and 5 mm × 5 mm fruit [7,8]. It can grow as a small tree or shrub of 3 m or as a tree that reaches a maximum height of 35 m, depending on the region, as shown in Figure 1 [9]. *Ptaeroxylon obliquum* is distributed across the southern regions of Africa in countries including Angola, Kenya, Mozambique, South Africa, and Zimbabwe [10]. In South Africa, the distribution and uses of *P. obliquum* have been reported in most parts, including Eastern Cape, KwaZulu-Natal, Limpopo, Mpumalanga, and Western Cape province.

The availability of a particular plant species, economic factors, as well as specific cultural practices, influence the reliance on plants to mitigate life challenges. While *P. obliquum* has been used for building and structural support, it has also been widely reported for its medicinal uses in ethnobotany in treating both human and animal infections [11,12]. *Ptaeroxylon obliquum* has been used for a range of diseases, including rhinitis, rheumatism, tuberculosis, hypertension, wound healing, and headache [13]. Phytochemical analysis of *P. obliquum* has revealed its abundance in coumarins and chromones, and vast pharmacological activities such as antimicrobial activities, anti-inflammation, and anthelmintic activities [14]. Moreover, many interesting biological active compounds such as obliquumol isolated from *P. obliquum* leaves which has also been patented have been reported from this plant. In vitro and in vivo toxicity studies have also been reported in this widely used medicinal plant species. To date, there is no comprehensive review that summarized and discussed the advances made in this species despite the wide range of pharmacological activities reported, secondary metabolites isolated or identified and their use in ethnopharmacology. Therefore, the current study aims to review ethnomedicine, phytochemistry, pharmacology, in silico, and toxicological studies on *P. obliquum* and propose possible future research studies.

## 2. Methodology

Data was collected from electronic databases including Google Scholar, Science Direct, PubMed, Scopus, Web of Science, SciFinder, and Wiley Online. The material used as the source of information for this review includes articles, theses, and abstracts. The terms used to search are linked to *P. obliquum*, and included phytochemistry, isolated compounds, traditional uses, pharmacological activities, antimicrobial, antiproliferation, anti-inflammatory, anticancer, cytotoxicity, in silico studies, molecular dockings, genotoxicity, secondary metabolites, essential oils, antioxidants, antiparasitic, in vivo, and ex vivo.

### 2.1. Inclusion Criteria

- Published and peer-reviewed articles, theses, and published abstracts on traditional uses, phytochemistry, secondary metabolites, and pharmacological activities of *P. obliquum*.
- English full-text articles or of other languages with options for translation to English.
- All available data that included *P. obliquum*, prior to 31 January 2025.

### 2.2. Exclusion Criteria

- Non-English articles lacking comprehensive translations were not included (articles that require translation from other languages to English).
- Articles containing details about the plant, but beyond the scope of this review.

## 3. Results

### 3.1. Traditional Uses

As shown in Table 1, *P. obliquum* is a widely used plant in traditional medicine across various tribes of South Africa and other African countries. Reports have shown varying uses of different parts of the plant by specific South African groups. The use of *P. obliquum* as firewood, poles, and timber for building is a common practice by Xhosa people of Eastern Cape, South Africa [6,15,16]. This practice has not only been influenced by poverty or typical cultural customs, but also availability, strength, and length of the wood [11].

The application of *P. obliquum* as a traditional medicine encompasses both direct and indirect treatment of livestock. The direct method of treating livestock with *P. obliquum* involves the introduction of the bark into dipping containers, which aids in the removal of ticks. This approach is preferred by more than 25% of farmers [17,18]. The direct use on livestock is through the treatment of diseases such as diarrhea, intestinal parasite, and carbuncles [10,19,20]. In countries such as Angola, leaves are used to treat contagious pleuropneumonia in cattle and goats, while farmers in Eastern Cape (South Africa) use the leaves to control intestinal parasites in goats [12,21]. Additional ethnoveterinary uses of *P. obliquum* in South Africa include treatment of wounds using leaf decoction and crude bark mixed with oil [22,23].

The use of *P. obliquum* in the treatment of human diseases has been reported in the southern region of Africa. Ground roots are debarked and soaked in water for two days, then taken orally to treat hypertension by the Zulu tribe of northern Maputaland in KwaZulu-Natal, South Africa [24]. The leaves are chewed, and the juice is swallowed for the relief of toothaches, headaches, and in the treatment of hypertension [25]. Powdered wood is used by Xhosa people as snuff for recreation and for medical purposes to relieve headaches [8]. *Ptaeroxylon obliquum* was reported to be used by the Bapedi people of Limpopo province, in South Africa, for rhinitis and tuberculosis with a 100% fidelity level [26]. Treatment of respiratory diseases with *P. obliquum* was also corroborated by the study on Zulu people, which revealed the use of air-dried leaves soaked in menstruum (alcohol) for five days and the juice (extracts) where taken orally to treat tuberculosis and chest complaints [27]. *Ptaeroxylon obliquum* leaves decoction was taken orally by the people of Ga-Mashashane in the Limpopo province (South Africa) to treat diarrhea [28]. The reliance of traditional practices on plants is diverse and the particular uses of *P. obliquum* further emphasizes the importance of plants in human lives. It has been reported to be useful in male circumcision by some South African tribes (Ndebele, Pedi, Sotho, Tsonga, Tswana, Venda, and Xhosa), utilizing the leaves as a bandage to soothe pain and swelling after circumcision [29]. *Ptaeroxylon obliquum* also serves as a host for bracket fungi, referred to as isibindi in Xhoza, meaning liver. Isibindi is a cosmetic applied on the skin for social status, it is used by male initiates returning from initiation school and by women to treat skin imperfections [30].

**Table 1:** Traditional uses of different parts of *P. obliquum*.

Plant Part	Traditional Uses	References
Wood	Anthrax remedy for ticks in cattle	[31]
Bark	Bark is used to cure fevers, arthritis, and rheumatism. recreational and therapeutic remedy for headache relief. Cattle treatment for ticks	[22,23]
Leaves	Gastro-intestinal parasites, anthrax, myiasis, and wounds for goats. Crop diseases. For humans, it is used to treat headaches, hypertension, and toothaches. Rituals.	[11,12,23,25,30,32,33]
Bark, leaves	Myasis, wounds, removing body odor	[22,25,34,35]
Roots	Hypertension, arthritis, fever	[24,26]
Leaves, bark, stem, and roots	Livestock treatment for diarrhea, intestinal parasites, Newcastle, scabies, timpanism, wounds	[20]
Fodder	Contagious pleauropneumonia (cattle, goats)	[21]

### 3.2. Phytochemistry

#### 3.2.1. Phytochemical Analysis of *P. obliquum*

The presence of flavonoids presented as quercetin equivalent (QE) and phenolic content as gallic acid equivalents (GAE) content of *P. obliquum* leaf extracts extracted with ethanol and methanol (MeOH) were reported by Oyedemi et al. [36], with 62.73 mgQE/g for flavonoids and 275 mgGAE/g for phenols. These findings were significantly higher than 29.17 mgQE/g and 155 mgGAE/g reported by Moyo and Masika [22]. The presence of phenolic content in *P. obliquum* MeOH and water extracts was also corroborated by Soyelu and Masika [22]. The presence of saponin has also been reported on *P. obliquum* bark extracts at concentrations of 17.28 ± 0.76 mg/g [34]. Other secondary metabolites from *P. obliquum* include saptaeroxy, pyrogall, resins, and alkaloids [2,19,34].

#### 3.2.2. Essential Oils Identified from *P. obliquum*

*Ptaeroxylon obliquum* is known to be abundant in secondary metabolites such as chromones and coumarins, from which most of the reported active biological compounds are isolated. *Ptaeroxylon obliquum* is an important source of essential oils composed of monoterpene hydrocarbons (16.7%), sesquiterpene hydrocarbons (33.5%), and oxygenated sesquiterpenes (25.9%) [37]. These essential oils are listed in Table 2, with their respective molecular formulas analyzed using gas chromatography–mass spectrometry (GC-MS) equipped with a mass spectrometer Shimadzu GCMS-QP2010-Ultra Mass Detector (electron ionization, 70 eV, Kyoto, Japan). Collectively, essential oils contained by *P. obliquum* include bicyclogermacrene (**1**), 10-epi-elemol (**2**), caryophyllene (**3**), α-pinene (**4**), β-pinene (**5**), α-gurjunene (**6**), caryophyllene oxide (**7**) and small amounts of camphene (**8**), limonene (**9**), (-)-*cis-*β-elemene (**10**), α-cubebene (**11**), copaene (**12**), β-bourbonene (**13**), β-elemene (**14**), (-)-β-copaene isomer (**15**), (+)-aromadendrene (**16**), α-humulene (**17**), neoalloocimene (**18**), eudesma-3,7-(11)-diene (**19**), germacrene-D (**20**), (+)-β-selinene (**21**), γ-Cadinene (**22**), δ-cadinene (**23**), guaiol (**24**), humulene epoxide (**25**), α-eudesmol (**26**), τ-muurolol (**27**), and neointermedeol (**28**) [37].

#### 3.2.3. Isolated and Tentatively Identified Compounds from *P. obliquum*

As shown in Table 2 and Figure S1 (Supplementary data), different bioactive compounds were isolated or tentatively identified from different parts of *P. obliquum*. Mulholland et al. [2] assessed the chemistry of *P. obliquum* and reported the presence of ptaeroxylinol acetate (**29**) isolated from the roots. Studies on *P. obliquum* bark extracts reported that it contains bioactive compounds such as peucenin (**30**) and prenyletin (**31**) [2,38]. Previous work on *P. obliquum* heartwood (timber) extracts have resulted in the isolation of bioactive compounds such as scopoletin (**32**), ptaerobliquol (**33**) nieshoutin or cyclo-obliquetin (**34**), nieshoutol (**35**), obliquetin (**36**), aesculetin (**37**), obliquin (**38**), umtatin (**39**), heteropeucenin 7-methyl ether (**40**), heteropeucenin (**41**), heteropeucenin dimethyl ether (**42**), alloptaeroxylin (**43**), ptaerochromenol (**44**), peucenin 7-methyl ether (**45**), dehydroptaeroxylin (**46**), ptaeroglycol (**47**), ptaerocyclin (**48**), ptaeroxylone (**49**), obliquol (**50**), obliquetol (**51**), and ptaeroxylin (desoxykarenin) (**52**), as summarized in Table 3 [5,38,39,40,41,42]. Leaves of *P. obliquum* contain interesting bioactive compounds such as obliquumol (**53**), β-amyrin (**54**) and lupeol mixture (**55**), eranthin (**56**), and O-methylalloptaeroxylin (**57**) [43,44,45,46,47,48]. Compounds available in *P. obliquum* sterm bark include guaia-1(10),11-diene (**58**), gamma-gurjuneneperoxide-(2) (**59**), bicyclo[5.2.0]nonane, 2-methylene-4,8,8-trim ethyl-4-vinyl-(**60**), spathulenol (**61**), epiglobulol (**62**), cycloheptane, 4-methylene-1-methyl-2-(2-methyl-1-propen-1-y1)-1-vinyl (**63**), gigantol (**64**), cyclohexane, 1-ethenyl-1-methyl-2,4-bis(1-methylethenyl)-, [1S-(1α,2β,4β)]-(**65**), 1,5,9-cyclotetradecatriene, 1,5,9-trim ethyl-12-(1-methylethenyl)-(**66**), thunbergol (**67**), n-hexadecanoic acid (**68**), 9,12,15-octadecatrienoic acid,2,3-dihydroxypropyl ester, (Z,Z,Z)-(**69**), vaccenic acid, cis-(**70**), octadecanoic acid,2-[2-[2-(2-hydroxyethoxy) ethoxy] ethyl ester (**71**), hexadecenoic acid, ethyl ester (**72**), isopropyl linoleate (**73**), 7-hexadecenal, (Z)-(**74**), phenol, 2,5-bis(1,1-dimethyl ethyl)-(**75**), 1,3,6,10-cyclotetradecatetraene,3,7,11-trimethyl-14-(1-methylethyl)-(**76**), dodecane, 1-fluoro-(**77**), hentriacontane (**78**), sulfurous acid, 2-ethylhexyl hexadecyl ester (**79**), and hexacosyl acetate (**80**) [36,49].

### 3.3. Pharmacological Activities

The reported studies on different pharmacological activities of *P. obliquum* extracts, fractions and bioactive compounds are presented in Table 4. Pharmacological activities such as antibacterial, antimycobacterial, antifungal, antioxidant, anticholinesterase, genotoxicity, antiparasitic, anti-inflammatory antiproliferative, antioxidant, and cytotoxicity were reported.

#### 3.3.1. Antibacterial Activity

*Ptaeroxylon obliquum* leaf, stem, and bark extracts have been investigated by Nielsen et al. [56] for their antibacterial effects against *Citrobacter*, *Escherichia coli*, *Klebsiella pneumoniae*, and *Pseudomonas aeruginosa*. The results highlighted that there were no significant antibacterial effects from leaf and stem extracts with minimum inhibitory concentrations (MIC) above 150 µg/mL; however, an MIC of 78 µg/mL was obtained on bark extracts against *E. coli*. Oyedemi et al. [36] tested the bactericidal effects of acetone, chloroform, ethanol, and MeOH leaf extracts of *P. obliquum* on *E. coli*, *Enterococcus faecalis*, *Proteus vulgaris*, *P. mirabilia*, *P. aeruginosa*, *Shigella sonnei*, and *Staphylococcus aureus*. The results showed selective effectiveness of extracts from different solvents on respective bacterial strains, with MICs ranging from 4 to 128 µg/mL. Acetone leaf extracts of *P. obliquum* from different geographical locations were also investigated by Ramadwa et al. [51] for antibacterial activities against *E. coli*, *E. faecalis*, *P. aeruginosa*, and *S. aureus,* were ranged from 100 to 320 µg/mL, and this was considered to be reasonable activity. Moreover, Ramadwa et al. [47] extended the investigation to *P. obliquum* with fractions and obliquumol (**53**). The results revealed that n-hexane fractions were the most active, with MICs ranging from 20 to 160 µg/mL, and obliquumol (**53**) had antibacterial activity against *P. aeruginosa* and *S. aureus* with an MIC of 31.5 µg/mL for both bacterial strains. In search of an anthrax remedy, Famuyide et al. [57] investigated antibacterial and antibiofilm activities of the bark and leaf crude extracts of *P. obliquum* against the *Bacillus anthracis Sterne* vaccine strain. The MICs of acetone and chloroform crude extracts from *P. obliquum* for biofilm suppression ranged from 0.005 to 0.039 mg/mL, with acetone bark and chloroform leaf extracts being the most active. The best antibacterial activity was also shown by acetone bark extracts. In another study, the antibacterial potential of *P. obliquum* leaf and bark extracts was tested in a study of formulating medicinal plant-based soap [58]. The study involved testing antimicrobial activities on Gram-positive bacteria (*Brevibacterium agri*, *B. mepidermidis*, *B. linens*, *Corynebacterium xerosis*, *Cutibacterium acnes*, *S. aureus*, *S. capitis*, *S. epidermidis*, *S. haemolyticus*, and *S. lugdunensis*) and Gram-negative bacteria *Acinetobacter baumanii*, *Enterobacter cloacae*, *E. coli*, *K. pneumoniae*, and *P. aeruginosa*. The best antibacterial activities in the study were shown by *P. obliquum* bark extracts on *B. linens*, *C. acnes*, *C. xerosis*, *P. aeruginosa*, and *S. aureus* with MIC values less than 0.50 mg/mL.

#### 3.3.2. Antifungal Activity

Nielsen et al. [56] assessed the antifungal activities of *P. obliquum* leaf, stem, and bark extracts against two fungi species, *Candida albicans* and *Microsporum audouinii*. The antifungal activities of the *P. obliquum*–methanol crude extracts had MIC values ranging from 78.12 µg/mL to 312.50 µg/mL. However, the antifungal activity of *P. obliquum* was reported by Nielsen et al. [56]. Van Wyk et al. [53] further investigated the antifungal activities of bioactive compounds available in *P. obliquum* acetone leaf extracts and fractions against *C. albicans* strains. The results revealed that fractions expressed varying susceptibility to *C. albicans* isolates with MICs ranging from 22 µg/mL to 740 µg/mL, with chloroform fractions being the most active with 22 µg/mL against *C. albicans*. These findings motivated the isolation and characterization of antifungal compounds, to which two commonly found compounds, β-amyrin (**54**) and lupeol (**55**) mixture eranthin (**56**), were isolated and one unknown pure compound, O-acetyl derivative which was identified as 8,11-dihydro-5-hydroxy-12-hydroxymethyl-2-methyl-4H-pyrano [2,3-g] [1] benzoxepin-4-one 12-O-acetate and named obliquumol (**53**). The effects of *P. obliquum* against *C. albicans* were also demonstrated by inhibiting the adherence of *C. albicans* to human buccal epithelial cells [59]. Ramadwa et al. [47] tested the β-amyrin (**54**) and lupeol (**55**) mixture, and obliquumol for antifungal activity against three opportunistic fungi, *C. albicans*, *Cryptococcus neoformans*, *Aspergillus fumigatus*, and *C. albicans* ATCC 10231. The findings suggested that the β-amyrin (**54**) and lupeol (**55**) mixture had significant antifungal activity against *C. neoformans* and *C. albicans* ATCC 10231 MICs of 16 µg/mL on both fungi. Moreover, obliquumol (**53**) had interesting results against *C. neoformans*, *A. fumigatus,* and *C. albicans* ATCC 10231 with the MICs of 2 µg/mL, 8 µg/mL, and 16 µg/mL, respectively. Antifungal activities of *P. obliquum* acetone crude leaf extract, fractions (hexane, chloroform, butanol, water/MeOH, and water) and isolated compounds (obliquumol, and lupeol and β-amyrin mixture) were further tested against phytopathogenic fungal species (*Aspergillus niger*, *A. parasiticus*, *Colletotrichum gloeoporioides*, *Fusarium oxysporum*, *Penicillium digitatum*, *P. expansum*, *P. italicum*, *P. janthinellum*, and *Rhizoctonia solani*) [53]. The results obtained indicated that the crude extracts exhibited MICs ranging from 80 µg/mL to 1250 µg/mL, with only hexane fractions showing promising effectiveness against *A. niger* and *P. digitatum* with the MIC values of 80 µg/mL for both, and *Rhizoctonia solani* was the most susceptible to the compounds with the MIC values of obliquumol (**53**) and, the β-amyrin (**54**) and lupeol (**55**) mixture at 8 µg/mL and 16 µg/mL, respectively. The antifungal activity of the compounds was also seen on plant fungal pathogens such as *A. niger*, *C. gloeoporioides*, *P. digitatum*, and *P. expansum* with the MICs as low as 32 µg/mL. Semi-synthesized ptaeroxylinol was assessed for its antifungal potential [51]. The compound had significant antifungal activities on the standard *C. albicans* ATCC 10.231 and *C. neoformans*, with MIC values of 8 µg/mL and 16 µg/mL, respectively. However, it had less effect on *A. fumigatus* and another *C. albicans* strain with MIC values of 62.5 and 31.5 µg/mL, respectively.

#### 3.3.3. Antimycobacterial Activities

*Mycobacterium bovis* (BCG P1172), *M. smegmatis* (ATCC 1441), *M. aurum* (NCTC 10437), and *M. fortuitum* (ATCC 6841) were used to determine antimycobacterial activities of *P. obliquum* acetone leaf extracts (butanol, n-hexane, chloroform, 30% H_2_O in MeOH, and water fractions), and isolated compounds (β-amyrin (**54**) and lupeol (**55**) mixture, and obliquumol (**53**) [47]. *Mycobacterium fortuitum* was the most susceptible to crude extracts and n-hexane, both with an MIC of 20 µg/mL, and even better antimycobacterial activities were shown on obliquumol, with an MIC of 8 µg/mL. Obliquumol (**53**) had relatively good antimycobacterial activity with an MIC of 16 µg/mL against *M. smegmatis*. In a separate study, acetone crude extracts, fractions, and isolated compounds from *P. obliquum* were evaluated for antimycobacterial activity against pathogenic *Mycobacterium tuberculosis* (ATCC 25177) and *Mycobacterium bovis* (ATCC 27290). Obliquumol (**53**) was reported to have the lowest MIC of 63 µg/mL on *M. tuberculosis* [14]. The β-amyrin (**54**) and lupeol (**55**) mixture had MICs of 125 µg/mL for the two pathogenic *mycobacterium strains* tested. Semi-synthesized ptaeroxylinol from isolated obliquumol from *P. obliquum* leaves was investigated for antimycobacterial activity against *M. aurum*, *M. bovis*, *M. fortuitum*, and *M. smegmatis*, and the MICs obtained were 250 µg/mL, 62.5 µg/mL, 250 µg/mL, and 62.5 µg/mL, respectively [51].

#### 3.3.4. Antioxidant Activities

Khunoana et al. [48] investigated the antioxidant activities of *P. obliquum* (cold/hot water, and acetone) leaf extracts, and chloroform and hexane fractions using 1,1-diphenyl-2-picrylhydrazyl (DPPH) assay and 2,2′-azinobis(3-ethylbenzothiazoline-6-sulfoxide) (ABTS+). Varying results were obtained between the two methods across crude extracts and fractions, with acetone extracts and fractions showing negligible free radical scavenging activities with IC_50_ values ranging from 8.9 µg/mL to 333.2 µg/mL and 3.3 µg/mL to 423.5 µg/mL, respectively. However, water extracts showed good antioxidant activity on ABTS, with an IC_50_ value of 21.5 µg/mL at the lowest concentration (3.125 µg/mL). The results were in contrast with the results previously obtained by Oyedemi et al. [36], particularly on *P. obliquum* acetone extracts, where strong free radical scavenging activities on DPPH were observed, with an IC_50_ value of 41 µg/mL. This might be due to different geographical locations where the leaves were collected, which might have an impact on the concentration of secondary metabolites [48].

#### 3.3.5. Anti-Inflammatory Activities

Ramadwa et al. [50] investigated the anti-inflammatory activities of acetone *P. obliquum* leaf extracts, fractions, and isolated compounds. Acetone *P. obliquum* leaf extracts and five fractions had weak inhibitory activity on the key leukotrienes regulator, lipoxygenase homolog (15-LOX), with IC_50_ ˃ 1.61 mg/mL. Isolated compounds eranthin (**56**), β-amyrin (**54**) and lupeol (**55**) mixture, and obliquumol (**53**) exhibited good inhibitory activities against 15-LOX with IC_50_ values of 0.0075 mg/mL, 0.0074 mg/mL, and 0.0139 mg/mL, respectively. The study also explored the anti-inflammatory activities of acetone *P. obliquum* leaf extracts, fractions, and isolated compounds on lipopolysaccharide (LPS)-induced macrophages RAW264.7 nitric oxide (NO), a key regulator of an inflammatory-related disease called rheumatoid arthritis. The results obtained indicated that there was a high percentage of NO inhibition. However, the high percentage inhibitions were associated with the toxicity of crude extracts, fractions, and isolated compounds on macrophages, with the highest viability at 66.8%. As important inflammatory mediators, interleukins, interleukin 1 beta (IL-1β), IL-6, and tumor necrosis factor alpha (TNF-α) were subjected to *P. obliquum* aqueous and ethanol extracts for anti-inflammation evaluation [60]. The outcome of the study revealed that the extracts had good anti-inflammatory activities and significantly inhibited IL-1β and IL-6. Although little inhibition of TNF-α occurred on aqueous extracts, ethanol extracts showed inhibition. These promising reports on anti-inflammatory activities validate the previous outcomes by McGaw et al. [61] on investigating the potential of *P. obliquum* wood aqueous and ethanol extracts to reduce inflammation through inhibiting a key inflammatory mediator called cyclooxygenase, based on ethnobotanical use of *P. obliquum* to treat headache. In this study, MeOH extracts were the most potent, with 61% cyclooxygenase inhibition at the concentration of 5 µg/mL.

#### 3.3.6. Antiparasitic Activities

Maharaj et al. [62] tested the adulticidal activity of the leaf, stem, and root of *P. obliquum* extracted with dichloromethane (DCM), DCM–MeOH, MeOH, and water. *Anopheles gambiae* and *An. arabiensis* were subjected to the aforementioned treatments. The results obtained suggested that different plant parts had varying effects, with *An. arabiensis* showing susceptibility to the water leaf extracts with 57% mortality, followed by stem extracts of the same solvent with 33%, then roots with 27% mortality. These results further elaborate on the impact of using different extracting solvents, as the mortality observed from organic solvents of the leaf extracts was only 7%. *Ptaeroxylon obliquum* acetone crude leaf extract isolated compounds (obliquumol) were tested for anthelmintic activities against a known livestock parasite, *Haemonchus contortus*. Good anthelmintic activities were yielded on crude extracts against the egg hatch and the larval development tests with LC_50_ values of 3.08 mg/mL and 2.21 mg/mL, respectively. Interestingly, obliquumol (**53**) was significantly potent to both the egg hatch and the developing larva with LC_50_ values of 0.22 mg/mL and 0.095 mg/mL [14]. The results motivated the investigation of *P. obliquum* fractions (hexane, chloroform, butanol, water/MeOH, and water) and isolated compounds (obliquumol (**53**), and the lupeol (**55**) and β-amyrin (**54**) mixture) on phytopathogenic parasite, *meloidogyne incognita (J2)*. The results obtained showed that the lupeol (**55**) and β-amyrin (**54**) mixture had a significant inhibition at 0.8 mg/mL and 1.0 mg/mL [53]. In a study by Moyo and Masika [22], bark water extracts of 40% *P. obliquum* reduced tick loads of *Rhipicephalus appendiculatus* and *Rhipicephalus microplus* by 26.8% and 11%, respectively. The acaricidal effects of *P. obliquum* bark extracts were scientifically tested and repelled 98% of ticks in 40 min at 40% concentration; this showed significant tick-repelling activities compared to tabard (positive control), which showed total repellence on the 7th hour at 35% concentration [19,34]. Antiparasitic activities of DCM *P. obliquum* extracts have also been reported on human disease-causing parasites, which include *Plasmodium falciparum*, *Trypanosoma cruzi*, *T. brucei rhodesiense*, and *Leishmania donovani* [63], and the IC_50_ obtained were 10.9 µg/mL, 41.5 µg/mL, 11.3 µg/mL, and 17.2 µg/mL, respectively. The *P. falciparum* findings on DCM *P. obliquum* extracts agreed with the previously reported antimalarial activity of *P. obliquum*, with IC_50_ values of 19 µg/mL, 19.5 µg/mL, and 11.5 µg/mL for roots, leaves, and stems, respectively [55]. The IC_50_ values of water extracts were ˃100 µg/mL across all plant parts; however, DCM/MeOH roots, leaves, and stem extracts showed good antiplasmodial activity compared to other extracts with IC_50_ values of 17 µg/mL, 22.8 µg/mL, and 5.5 µg/mL, respectively. Ptaerobliquol (**33**) isolated from *P. obliquum* roots was tested for antiparasitic activity against Toxoplasma gondii was investigated and had moderate activity with an IC_50_ of 5.13 µM [38].

#### 3.3.7. In Silico Studies

Ojo et al. [49] conducted an assessment, predicting inhibitory activities of *P. obliquum* compounds derived from GC-MS on proteins (acetylcholinesterase, butyrylcholinesterase, and β-secretase) involved in the pathogenesis of Alzheimer’s disease through computational molecular docking, specifically using Autodock Vina on Pyrx virtual screening tool. The most potent inhibitors were cyclotetradecatriene (**66**) against all the selected proteins (1vot, 1xs7, and 7aiy) with binding affinities of −9.2 kcal/mol^−1^, −8.8 kcal/mol^−1^, and −8.7 kcal/mol^−1^, respectively. Thunbergol (**67**) also had strong binding affinity across all selected proteins with binding affinities of −8.4 kcal/mol^−1^, −8.4 kcal/mol^−1^, and −8.3 kcal/mol^−1^ on the selected proteins (1vot, 1xs7, and 7aiy). Moreover, it is noteworthy that certain substances, including spathulenol (**61**), epiglobulol (**62**), guaia-1(10),11-diene (**58**), gamma-gurjunenepoxide-(2), 8-(1,1-dimethylallyl)-5,7-dimethoxy coumarin (**59**), and bicyclo [5.2.0] nonane, 2-methylene-4,8,8-trimethyl-4-vinyl (**60**), showed high binding affinity, but they are specific to certain proteins or receptors.

#### 3.3.8. Anti-Cholinesterase Activities

Ojo et al. [49] assessed the in vitro inhibitory activities of the *P. obliquum* crude extracts against acetylcholinesterase and butyrylcholinesterase. The results showed that the dichloromethane (DCM) extract of *P. obliquum* had the highest butyrylcholinesterase inhibitory activity with an IC_50_ value of 1.77 µg/mL. Hexane extracts had good butyrylcholinesterase activity with an IC_50_ value of 4.79 µg/mL, while ethanol extract had 3.54 µg/mL. The inhibitory activities of the crude extracts against acetylcholinesterase were generally low. DCM extracts had an IC_50_ value of only 66.59 µg/mL, while the other two, hexane and ethanol, had IC_50_ values of 77.01 µg/mL and 69.05 µg/mL, respectively. The β-secretase activity inhibition results demonstrated that the crude extracts possessed β-secretase inhibitory potential, though the hexane crude extract of *P. obliquum* had the lowest IC_50_ value of 29.5 µg/mL, while DCM and ethanol extracts had IC_50_ values of 41.1 µg/mL and 35.5 µg/mL, respectively. However, there was no significant difference in their β-secretase inhibition activity. The β-secretase inhibitory activity of the crude extracts was evaluated using a commercial assay kit (CS0010-1KT, Sigma, MO, USA) following the manufacturer’s instructions. The Thioflavin-T assay was used to evaluate Aβ aggregation and disaggregation properties of the crude extracts. The result of *P. obliquum* was not included because the extracts showed no significant Aβ1-42 aggregation attenuation activity.

#### 3.3.9. In Vivo Studies

In vivo studies were carried out on female Swiss albino mice/CDI to determine acute animal toxicity of obliquumol (**53**) according to OECD 423 guidelines (Ramadwa et al., [49]. The study involved the administration of obliquumol orally, regularly monitoring, weighing the mice, and lastly, the mice were subjected to necropsy and histopathology examination. The post-mortem observation indicated that obliquumol (**53**)-treated mice had all intact, normal-sized livers, lungs well aerated, hearts in good condition, and all the organs had normal colors. The histopathology report showed that the livers had evidence of mild, sublethal, non-specific hepatocellular injury, and there were background lesions such as nephritis; however, there was no sign of necrosis or inflammation. Obliquumol had an LD_50_ > 2000 mg/kg since there were no mortalities after 14 days.

### 3.4. Toxicological Studies

#### 3.4.1. Cytotoxicity

Cytotoxicity of *P. obliquum* has been reported on various human and animal cell lines. Following antifungal activities of *P. obliquum* leaf extracts, Van Wyk et al. [46] investigated the safety of acetone *P. obliquum* leaf extracts through assessing cytotoxicity effects on mouse fibroblast cells, and the result obtained showed moderate toxicity at an LC_50_ value of 35.6 µg/mL. Ramadwa et al. [14] have reported the cytotoxicity effects of acetone *P. obliquum* leaf crude extracts on monkey Vero cells and human liver (C3A) cells, with significant toxicity at a value of CC_50_ 14.2 µg/mL for Vero cells and low toxicity at 106.5 µg/mL for C3A cells. This finding was corroborated through an in vitro cytotoxic assessment of acetone *P. obliquum* crude extracts by Khunoana et al. [48], where proliferation of Vero cells was inhibited at the lowest IC_50_ value of 4.5 µg/mL. In the same study, they also investigated the cytotoxicity through subjecting Vero cells, human liver hepatocarcinoma (HepG2) cells, human breast adenocarcinoma (MCF-7), human cervical cancer (HeLa) cells, and human lung adenocarcinoma (A549) cells to a range concentrations of cold and hot water *P. obliquum* crude extracts from the plant collected from different areas. The results showed that different geographical locations have varying effects on the cytotoxicity activities of *P. obliquum* extracts and lowest IC_50_ value ranges against Vero cells, HepG2 cells, MCF-7 cells, HeLa cells, and A549 cells were, respectively, as follows: hot H_2_O at 322.5 ± 15.1 µg/mL, and cold H_2_O 449.5 ± 0.8 µg/mL; acetone 8.6 ± 0.2 µg/mL, hot H_2_O at 607 ± 8.3 µg/mL, and cold H_2_O at 246 ± 4.6 µg/mL; acetone at 23.3 ± 6.6 µg/mL, hot H_2_O at 418.7 ± 109.6 µg/mL, and cold H_2_O at 487.8 ± 11.9 µg/mL; acetone at 34.8 ± 6.9 µg/mL, hot H_2_O at 694.5 ± 56.6 µg/mL, and cold H_2_O at 820.4 ± 104.9 µg/mL; acetone at 64.1 ± 8.4 µg/mL, hot H_2_O at 136.6 ± 17.8 µg/mL, and cold H_2_O at 188.7 ± 12.3 µg/mL. Ramadwa et al. [50] further assessed the cytotoxicity effects of acetone *P. obliquum* leaf extracts on macrophage (RAW 264.7) cells. The cells were subjected to different concentrations (2 µg/mg, 10 µg/mg, 30 µg/mg, and 100 µg/mg), and the cell viability of 50.2%, 48.5%, 47.6%, and 1.7%, respectively. In an antiproliferation test for semi-synthesized ptaeroxylinol against Vero cells and C3A, the result obtained suggested that the compound was not toxic to normal cells, with IC_50_ values of 85.7 µg/mL and 126.51 µg/mL, respectively.

#### 3.4.2. Genotoxicity

McGaw et al. [54] investigated the genotoxicity of *P. obliquum* acetone crude leaf extracts on *Salmonella typhimurium* tester strains, TA 98 and TA 100. The outcome suggested that the extracts had no mutagenic effects on both strains. The same was reported on *P. obliquum* acetone crude leaf extracts, (butanol, n-hexane, chloroform, 30% H_2_O in methanol, and water) fractions, and isolated compounds [the β-amyrin (**54**) and lupeol (**55**) mixture, and obliquumol (**53**)] were tested for genotoxicity against S. typhimurium tester strains, TA 98, TA 100, and TA 102 using the Ames test [53]. The crude extracts, fractions, and isolated compounds had no genotoxic effects against all *S. typhimurium* tester strains used in the study.

## 4. Discussion and Future Perspectives

*Ptaeroxylon obliquum* is distributed across southern Africa, in countries such as Angola, Kenya, Mozambique, South Africa, and Zimbabwe. The common name for *P. obliquum* is sneezewood, a name given due to its potent irritation properties that were later associated with its abundance in coumarins and chromones. Reports have shown that different parts of the plant are used in tradition for purposes such as firewood, in construction, and most importantly as a medicinal plant for humans and as an ethnoveterinary medicine. Some of the reported traditional uses of *P. obliquum,* such as antiparasitic agent and medicine for livestock, have been scientifically tested.

**Figure 2:**
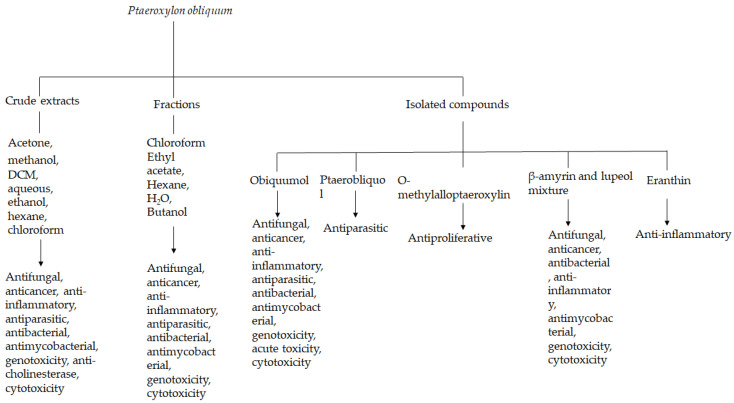
Schematic flow of *P. obliquum* constituents and their respective pharmacological activities.

*Ptaeroxylon obliquum* extracts have been shown to have antiparasitic properties against a variety of parasites, including *R. appendiculatus*, *R. microplus*, *H. contortus*, and *M. incognita*. This may explain and validate the use of different parts of *P. obliquum* in southern Africa against a range of endo and ectoparasites. The in vivo efficacy studies must, of course, validate the in vitro results. The crude extracts also had anti-inflammatory and antimycobacterial properties, even against human and animal pathogenic mycobacterium species, which justifies the use of the plant species to treat inflammatory-related conditions and tuberculosis. Several classes of compounds have been reported from this species, but it is clear that *P. obliquum* extracts are rich in chromones and coumarins. A total of 80 secondary metabolites from different classes of compounds have been reported from this plant species. Obliquumol and ptaerobliquol were isolated from nature for the first time from *P. obliquum* leaf and root extracts. The anti-cholinesterase activities and computational molecular docking predicting inhibitory activities of *P. obliquum* compounds tentatively identified by GC-MS on proteins involved in the pathogenesis of Alzheimer’s disease, which revealed that cyclotetradecatriene (**66**) and thunbergol (**67**) had strong binging affinity on proteins associated with Alzheimer’s disease further give credence to the use of the plant to treat Alzheimer related conditions. The schematic diagram (Figure 2) summarizes the pharmacological properties reported on the respective plant constituents, interestingly showing that of the 80 compounds reported in this review, only a few have been investigated. Future research should focus on the in vivo efficacy of *P. obliquum* and the most promising bioactive compounds. Perhaps the most important studies conducted on this plant species were the extensive pharmacological activities on obliquumol (**53**) and the many interesting biological activities reported, including antifungal, antimycobacterial, anti-inflammatory, and antiparasitic activities of the compound. Very few natural products are tested in in vivo studies due to low concentrations of compounds and variations that are influenced by many factors. However, in the case of obliquumol, a method was developed to isolate large quantities of the compound from *P. obliquum* leaf extracts, which made it possible to determine the in vivo acute toxicity of the compound [51,53]. The compound has also been chemically synthesized [64], which makes it a great candidate for future in vivo efficacy studies. Possible mechanisms of action should be considered, and the ADME pharmacological activities (absorption, distribution, metabolism, and excretion) could be useful to determine therapeutic potential and biodistribution in vivo animal models and validate the wide range of traditional uses of *P. obliquum*. Furthermore, obliquumol (**53**) isolated from *P. obliquum* is a potential therapeutic agent due to its lower toxicity demonstrated in both in vitro studies against several cell lines and in in vivo studies. Since the extensive in vitro pharmacological activities of *P. obliquum* extracts, future studies should focus on in vivo anti-inflammatory, antifungal, antiparasitic, and antimycobacterial activities.

## 5. Conclusions

*Ptaeroxylon obliquum* is an important plant which has been traditionally utilized for various purposes, including building, fencing, and as a traditional medicine. Ethnobotanical studies efficiently highlighted the importance of *P. obliquum* in treating different diseases in both humans and animals/livestock. This sparked interest in scientific studies, that revealed the phytochemical composition and pharmacological properties of *P. obliquum*. Coumarins and chromones are the most abundant secondary metabolites reported, and interesting bioactive compounds such as obliquumol have shown excellent antifungal activities and were not toxic in in vitro cytotoxicity, genotoxicity, and acute animal toxicity studies conducted. Other pharmacological activities, such as anti-inflammatory, antimicrobial, and antiparasitic properties reported on *P. obliquum* crude extracts, fractions, and isolated compounds, showed the potential of the plant to treat different diseases. The diversity in the ethnobotanical uses of *P. obliquum* and pharmacological activities comprehensively summarized in this review suggests that the plant has the potential to have multitherapeutic properties, and more research is necessary to broaden and deepen the understanding of the potential therapeutic activities of the plant and isolated compounds, especially in vivo animal models to understand the mechanisms of actions. This review thoroughly captures the most relevant and valuable aspects of *P. obliquum* and its constituents, directly contributing to the growing global focus on phytotherapy and encouraging preservation of the medicinal plant species.

## Figures and Tables

**Table 2:** Chemical composition of the essential oils from *P. obliquum*.

No.	Compounds	Molecular Formula	Molecular Weight (g/mol)	Area (%)	Retention Time
**1**	Bicyclogermacrene	C_15_H_24_	204.35	7.9	15.24
**2**	10-Epi-elemol	C_15_H_26_O	222.37	7.3	16.31
**3**	Caryophyllene	C_15_H_24_	204.35	6.8	13.61
**4**	α-Pinene	C_10_H_16_	136.23	6.0	3.82
**5**	β-Pinene	C_10_H_16_	136.23	5.1	4.42
**6**	α-Gurjunene	C_15_H_24_	204.35	1.3	13.37
**7**	Caryophyllene oxide	C_15_H_24_O	220.35	0.4	17.06
**8**	Camphene	C_10_H_16_	136.23	4.4	4.03
**9**	Limonene	C_10_H_16_	136.23	1.2	5.22
**10**	(-)-*cis*-β-Elemene	C_15_H_24_	204.35	0.6	11.72
**11**	α-Cubebene	C_15_H_24_	204.35	0.3	12.03
**12**	Copaene	C_15_H_24_	204.35	1.2	12.61
**13**	β-Bourbonene	C_15_H_24_	204.35	0.4	12.82
**14**	β-Elemene	C_15_H_24_	204.35	0.6	12.93
**15**	(-)-β-Copaene isomer	C_15_H_24_	204.35	0.7	13.79
**16**	(+)-Bromadendrene	C_15_H_24_	204.35	1.2	14.02
**17**	α-Humulene	C_15_H_24_	204.35	2.1	14.35
**18**	Neoalloocimene	C_10_H_16_	136.23	0.5	14.51
**19**	Eudesma-3,7-(11)-diene	C_15_H_24_	204.35	1.4	14.78
**20**	Germacrene-D	C_15_H_24_	204.35	3.5	14.91
**21**	(+)-β-Selinene	C_15_H_24_	204.35	0.4	15.05
**22**	ץ-Cadinene	C_15_H_26_	204.35	1.5	15.59
**23**	δ-Cadinene	C_15_H_24_	204.35	4.1	15.76
**24**	Guaiol	C_15_H_26_O	222.35	3.1	17.32
**25**	Humulene epoxide	C_15_H_24_O	220.35	0.3	17.59
**26**	α-Eudesmol	C_15_H_26_O	222.35	4.5	18.01
**27**	τ-Muurolol	C_15_H_26_O	222.35	3.1	18.28
**28**	Neointermedeol	C_15_H_26_O	222.35	2.9	18.46

**Table 3:** Isolated or tentatively identified compounds from the different parts of *P. obliquum*.

No.	Compounds	Plant Part	Detection/Isolation Method	References
**29**	Ptaeroxylinol acetate	Roots	Isolated; IR, NMR	[2,38]
**30**	Peucenin	Heartwood or bark, roots	Isolated; HPLC, NMR	[2,38]
**31**	Prenyletin	Heartwood or bark, roots	Isolated; HPLC NMR	[38,39]
**32**	Scopoletin	Heartwood, roots	Isolated; HPLC, NMR	[2,39]
**33**	Ptaerobliquol	Heartwood	Isolated; Crystal X-ray analysis, UV, IR, NMR	[38]
**34**	Nieshoutin/Cyclo-obliquetin	Heartwood	Isolated; UV, NMR	[39,40]
**35**	Nieshoutol	Heartwood	Isolated; UV, NMR	[39,40]
**36**	Obliquetin	Heartwood	Isolated; NMR	[38,39]
**37**	Aesculetin	Heartwood	Isolated; UV, NMR	[40]
**38**	Obliquin	Heartwood	Isolated; NMR	[39,40]
**39**	Umtatin	Heartwood	Isolated; NMR	[5,39]
**40**	Heteropeucenin 7-methyl ether	Heartwood	Isolated; NMR	[39]
**41**	Heteropeucenin	Wood	Isolated; UV, NMR	[39]
**42**	Heteropeucenin dimethyl ether	Wood	Isolated; UV, NMR	[39]
**43**	Alloptaeroxylin	Wood	Isolated; UV, NMR	[39]
**44**	Ptaerochromenol	Wood	Isolated; UV, NMR	[39]
**45**	Peucenin 7-methyl ether	Wood	Isolated; UV, NMR	[39]
**46**	Dehydroptaeroxylin	Wood	Isolated; UV, NMR	[39]
**47**	Ptaeroglycol	Wood	Isolated; UV, NMR	[39]
**48**	Ptaerocyclin	Wood	Isolated; UV, NMR	[39]
**49**	Ptaeroxylone	Wood	Isolated; UV, NMR	[39]
**50**	Obliquol	Heartwood	Isolated; NMR	[5,39]
**51**	Obliquetol	Wood	Isolated; UV, NMR	[39]
**52**	Ptaeroxylin (desoxykarenin)	Heartwood	Isolated; NMR, X-ray	[5,39,42]
**53**	Obliquumol	Leaves	Isolated; NMR, UPLC-MS	[46,48,50,51]
**54**	β-Amyrin	Leaves	Isolated; NMR	[46,48,50,51]
**55**	Lupeol	Leaves	Isolated; NMR	[46,47]
**56**	Eranthin	Leaves	Isolated; NMR	[42,47,52]
**57**	*O*-Methylalloptaeroxylin	Leaves	Isolated; NMR	[43,44,45,48]
**58**	Guaia-1(10),11-diene	Stem bark	Tentatively identified; GC-MS	[49]
**59**	Gamma-Gurjuneneperoxide-(2)	Stem bark	Tentatively identified; GC-MS	[49]
**60**	Bicyclo [5.2.0] nonane, 2-methylene-4,8,8-trim ethyl-4-vinyl-	Stem bark	Tentatively identified; GC-MS	[49]
**61**	Spathulenol	Stem bark	Tentatively identified; GC-MS	[49]
**62**	Epiglobulol	Stem bark	Tentatively identified; GC-MS	[49]
**63**	Cycloheptane, 4-methylene-1-methyl-2-(2-methyl-1-propen-1-y1)-1-vinyl-	Stem bark	Tentatively identified; GC-MS	[49]
**64**	Gigantol	Stem bark	Tentatively identified; GC-MS	[49]
**65**	Cyclohexane, 1-ethenyl-1-methyl-2,4-bis(1-methylethenyl)-, [1S-(1α,2β,4β)]-	Stem bark	Tentatively identified; GC-MS	[49]
**66**	1,5,9-Cyclotetradecatriene, 1,5,9-trim ethyl-12-(1-methylethenyl)-	Stem bark	Tentatively identified; GC-MS	[49]
**67**	Thunbergol	Stem bark	Tentatively identified; GC-MS	[49]
**68**	*n*-Hexadecanoic acid	Stem bark	Tentatively identified; GC-MS	[49]
**69**	9,12,15-Octadecatrienoic acid,2,3-dihydroxypropyl ester, (Z,Z,Z)-	Stem bark	Tentatively identified; GC-MS	[49]
**70**	Vaccenic acid, *cis-*	Stem bark	Tentatively identified; GC-MS	[49]
**71**	Octadecanoic acid,2-[2-[2-(2-hydroxyethoxy) ethoxy] ethyl ester	Stem bark	Tentatively identified; GC-MS	[49]
**72**	Hexadecenoic acid, ethyl ester	Stem bark	Tentatively identified; GC-MS	[49]
**73**	Isopropyl Linoleate	Stem bark	Tentatively identified; GC-MS	[49]
**74**	7-Hexadecenal, (Z)-	Stem bark	Tentatively identified; GC-MS	[49]
**75**	Phenol, 2,5-bis (1,1-dimethyl ethyl)-	Stem bark	Tentatively identified; GC-MS	[49]
**76**	1,3,6,10-Cyclotetradecatetraene,3,7,11-trimethyl-14-(1-methylethyl)-	Stem bark	Tentatively identified; GC-MS	[36]
**77**	Dodecane, 1-fluoro-	Stem bark	Tentatively identified; GC-MS	[36]
**78**	Hentriacontane	Stem bark	Tentatively identified; GC-MS	[36]
**79**	Sulfurous acid, 2-ethylhexyl hexadecyl ester	Stem bark	Tentatively identified; GC-MS	[36]
**80**	Hexacosyl acetate	Stem bark	Tentatively identified; GC-MS	[36]

Key: nuclear magnetic resonance (NMR); high performance liquid chromatography (HPLC); ultra-performance liquid chromatography–mass spectrometry (UPLC-MS); gas chromatography–mass spectrometry (GC-MS); ultraviolet (UV).

**Table 4:** Pharmacological activities of *P. obliquum* extracts, fractions, and isolated compounds.

Plant Part	Crude Extracts/Fractions/Compound	Pharmacological Activities	Bioassay Model	Results	References
Leaves	Acetone extracts	Antifungal	MIC	*A. niger*, *C. gloeosporioides* and *P. digitatum* had MICs of 80 μg/mL.	[49]
		Cytotoxicity	MTT	Toxic against Vero cells with CC_50_ = 14.2 μg/mL and IC_50_ of 16.1 μg/mL.	[14,48]
		Antibacterial	MIC	MIC of 4 µg/mL against *S. sonnei* and 16.4 µg/mL against *S. pneumoniae* and *P. mirabilis.*	[36]
		Anticancer	MTT	IC_50_ of 8.6 ± 0.8 µg/mL on HEG2 and 23.3 ± 6.6 µg/mL on MCF7.	[48]
		Antioxidant	DPPH ABTS	IC_50_ of 150.6 ± 1.2 µg/mL IC_50_ of 251.2 ± 50 µg/mL	
		Genotoxicity	Ames test	Non mutagenic against all tested strains *S. typhimurium* strains (TA98, TA100, and TA 102).	[53,54]
		Antimycobacterial	MIC	MIC of 20 μg/mL against *M. fortuitum.* MICs of ≤100 μg/mL against *M. aurum* and *M. bovi*	[14,47]
		Antiparasitic	Egg hatch assay (EHA), larval development test (LDT)	LC_50_ of 3.08 ± 0.05 mg/mL on EHA and 2.21 ± 0.18 mg/mL on LDT	[14]
	Aqueous extracts	Antiproliferative	MTT	IC_50_ of 136.6 ± 17.8 µg/mL against A549 cells.	[48]
		Antioxidant	DPPH ABTS	IC_50_ of 43.4 ± 6.1 µg/mL. IC_50_ of 21.5 ± 0.2 µg/mL	[48]
		Antiparasitic	Anti-plasmodial activity (*P. falciparum* D10)	IC_50_ of ˃100 µg/mL against *P. falciparum* D10.	[55]
		Antibacterial	MIC	MIC of 487 µg/mL against *P. mirabilis.*	[13]
	Chloroform fraction	Antifungal	MIC	MIC of 45 µg/mL against *C. albicans* strain.	[46]
		Anticancer	MTT assay	IC_50_ of 33.5 ± 3 µg/mL against HepG2.	[48]
		Antibacterial	MIC	MIC of 80 µg/mL against *P. aeruginosa*.	[47]
		Antioxidant	DPPH ABTS	IC_50_ of 387.4 ± 27.3 µg/mL IC_50_ of 214.2 ± 13.1 µg/mL	[48]
		Anti-inflammatory	15-LOX inhibition assay, NO inhibition assay	IC_50_ of 3.03 mg/mL on lipoxygenase enzyme	[50]
		Cytotoxicity	MTT	LC_50_ of 28.6 µg/mL against mouse fibroblast cells	[46]
		Genotoxicity	Ames test	Non mutagenic against *S. typhimurium* strains TA98, TA100, and TA102.	[53]
	Ethyl acetate fraction	Antifungal	MIC	MIC = 300 µg/mL on *C. albicans.*	[46]
		Cytotoxicity	MTT	IC_50_ of 229.7 µg/mL against fibroblast cells	
	Hexane fraction	Genotoxicity	Ames test	Not genotoxic on *S. typhimurium* strains (TA98, TA100, and TA 102) tested.	[53]
		Antioxidant	DPPH ABTS	IC_50_ = 236.5 ± 42.1 µg/mL IC_50_ = 143.7 ± 3.3 µg/mL.	[47]
		Antifungal	MIC	MIC = 180 µg/mL against *A. fumigatus.*	[14]
	H_2_O fraction	Antifungal	MIC	MIC = 2500.0 µg/mL against *A. fumigatus*, *A. niger*, *F. oxysporum* and *C. gloeosporioides*	[14,53]
		Cytotoxicity		LC_50_ of 0.08 µg/mL against mouse fibroblast cells.	[46]
	Butanol fraction	Antifungal	MIC	MIC of 320 µg/mL against *C. gloeosporioides* and 630 µg/mL against *A. niger*	[53]
		Antibacterial	MIC	MIC of 630 µg/mL of *S. aureus*	[47]
		Anti-inflammatory	15-LOX inhibition assay	IC_50_ of 6.55 mg/mL against 15-LOX enzyme.	[50]
	30% H_2_O–MeOH fraction	Antimycobacterial	MIC	MIC of 40 µg/mL against *M. fortuitum*.	[47]
		Antibacterial	MIC	MIC of 320 µg/mL against *S. aureus* and *E. faecalis.*	[47]
		Antifungal	MIC	MIC of 160 µg/mL against *A. fumigatus*	[47]
		Anti-inflammatory	15-LOX inhibition assay	IC_50_ of 5.24 mg/mL against 15-LOX enzyme.	[50]
		Cytotoxicity	MTT	CC_50_ of 49.6 ± 0.002 µg/mL against Vero cells.	[14]
		Genotoxicity	Ames test	Not genotoxic against *S. typhimurium* strains TA98, TA100, and TA 102.	[53]
	Obliquumol (**53**)	Antifungal	MIC	MIC of 2 µg/mL against *C. albicans* and 8 µg/mL against *C. neoformans*.	[14]
		Anticancer	MTT	IC_50_ of 52.7 ± 4.8 µg/mL against HepG2 cells	[48]
		Anti-inflammatory	15-LOX inhibition assay	IC_50_ = 1.39 µg/mL against 15-LOX enzyme.	[50]
		Antiparasitic	Egg hatch assay (EHA), larval development test (LDT)	LC_50_ of 0.22 ± 0.03 mg/mL against EHA and 0.095 ± 0.002 mg/mL against LDT on *H. contortus.*	[14]
		Antibacterial	MIC	MIC of 31.5 µg/mL against *S. aureus* and *P. aeruginosa.*	[47]
		Cytotoxicity	MTT	CC_50_ of ˃200 µg/mL against Vero and C3A cells. LC_50_ of 7.2 µg/mL against mouse fibroblast cells.	[46,47]
		Antimycobacterial	MIC	MIC of 8 µg/mL against *M. fortuitum* and 16 µg/mL against *M. smegmatis.* MIC of 63 µg/mL against pathogenic *M. tuberculosis* ATCC 25177	[14,46]
		Genotoxicity	Ames test	Not genotoxic against *S. typhimurium* strains TA98, TA100, and TA 102.	[53]
		In vivo animal studies	Acute toxicity (OECD 423 guidelines)	LD_50_ > 2000 mg/kg since no mouse mortalities occurred after 14 days.	[53]
	β-Amyrin (**54**) and lupeol (**55**) mixture	Antifungal	MIC	Lowest MIC of 16 µg/mL against *C. albicans* and *C. neoformans*.	[14]
		Anticancer	MTT	IC_50_ of 122.6 ± 1.8 µg/mL against HepG2 cells.	[48]
		Antibacterial	MIC	MIC of 62.5 µg/mL against *S. aureus* and *P. aeruginosa.*	[47]
		Anti-inflammatory	15-LOX inhibition assay	IC_50_ of 7.4 µg/mL against 15-LOX enzyme.	[51]
		Antimycobacterial	MIC	MIC of 62.5 µg/mL against *M. fortuitum* and *M. smegmatis.* MIC = 125 µg/mL against pathogenic *M. bovis* ATCC 27290 and *M. tuberculosis* ATCC 25177.	[14,47]
		Cytotoxicity	MTT	LC_50_ of 0.001 µg/mL against fibroblast cells.	[53]
		Genotoxicity	Ames test	Non mutagenic against *S. typhimurium* strains TA98, TA100, and TA 102.	[53]
	Eranthin (**56**)	Anti-inflammatory	15-LOX inhibition assay	LC_50_ = 7.5 µg/mL	[50]
	DCM extracts	Antiparasitic	Anti-plasmodial activity	IC_50_ = 19.5 µg/mL against *P. falciparum* D10.	[55]
	DCM–MeOH extracts	Antiparasitic	Anti-plasmodial activity	IC_50_ = 22.8 µg/mL against *P. falciparum* D10.	
	MeOH extracts	Antibacterial	MIC	MIC of 4 µg/mL against *S. sonnei* and 32 µg/mL against *S. aureus* and *P. vulgaris.*	[36]
		Antimycobacterial	MIC	MIC = ˃2500 µg/mL against *M. smegmatis*,	[56]
		Antifungal	MIC	MIC = 156.25 µg/mL against *C. albicans*	
		Antioxidant	DPPH	IC_50_ of ˂150 µg/mL	[36]
	O-methylalloptaeroxylin (**57**)	Antiproliferative	MTT	IC_50_ of 212.7 ± 1.8 µg/mL against HeLa cells and 151.5 ± 38.7 µg/mL on Vero cells.	[48]
	Ethanol extracts	Antibacterial	MIC	MIC of 4 µg/mL against *S. sonnei*.	[36]
	Chloroform extracts	Antibacterial	MIC	MIC = 8 µg/mL against *S. sonnei* and *P. mirabilis*	[36]
Stem	DCM–MeOH extracts	Antiparasitic	Antiplasmodial activity	IC_50_ = 5.5 µg/mL against *P. falciparum* D10.	[55]
	DCM extracts	Antiparasitic	Antiplasmodial activity	IC_50_ = 17 µg/mL against *P. falciparum* D10.	[55]
	Aqueous extracts	Antiparasitic	Antiplasmodial activity	IC_50_ > 100 µg/mL against *P. falciparum* D10.	[55]
Roots	DCM–MeOH extracts	Antiparasitic	Antiplasmodial activity	IC_50_ = 17 µg/mL against *P. falciparum* D10.	[55]
	DCM extracts	Antiparasitic	Antiplasmodial activity	IC_50_ = 19 µg/mL against *P. falciparum* D10.	
	Aqueous extracts	Antiparasitic	Antiplasmodial activity	IC_50_ > 100 µg/mL against *P. falciparum* D10.	
	Ptaerobliquol (**33**)	Antiparasitic	Antiplasmodial activity	*Toxoplasma gondii* had moderate activity with an IC_50_ of 5.13 µM	[38]
Bark	MeOH extracts	Antibacterial	MIC	MIC of 78.12 µg/mL against *E. coli.*	[56]
		Antifungal	MIC (*C. albicans* and *M. audouinii*)	MIC range from 78.12 µg/mL to 312.50 µg/mL	[56]
	Aqueous extracts	Antiparasitic	In vitro repellence and contact bio-assay models	A total of 40% reduction in *R. appendiculatus* and *R. microplus* by 26.8% and 11%, respectively	[22]
		Anti-inflammatory	MTT	Aqueous extracts significantly decreased (*p* < 0.0005) IL-6 and MCP-1 levels compared to the control.	[48]
	Ethanol extracts	Cytotoxicity	MTT	RAW 264.7 murine macrophages and human dermal fibroblasts had cell viability of >100% and >80%, respectively.	[48]
		Anti-inflammatory	MTT	Significantly decreased (*p* < 0.0005) IL-6 and TNF-α levels compared to the control.	[49]
Stem bark	Hexane extract	Anti-cholinesterase	Cholinesterase inhibitory activity assay	Had good butyrylcholinesterase activity with an IC_50_ of 4.79 µg/mL. Had acetylcholinesterase inhibitory activity with an IC_50_ of 77.01 µg/mL.	[49]
	DCM extracts	Anti-cholinesterase	Cholinesterase inhibitory activity assay	Had butyrylcholinesterase inhibitory activity with an IC_50_ of 1.77 µg/mL and acetylcholinesterase inhibitory activity of 66.59 µg/mL.	[49]

## Data Availability

Not applicable.

## Supplementary Materials

The following supporting information can be downloaded at: https://www.mdpi.com/article/10.3390/plants14121746/s1, Figure S1. Structures of the isolated or tentatively identified compounds from *Ptaeroxylon obliquum*.
