# Supplementary material for: A Review of the Ethnomedicine, Phytochemistry, Pharmacology and Toxicological Studies on Ptaeroxylon obliquum (Thunb.) Radlk. (Rutaceae)
Source: Plants (Basel). 2025 Jun 6;14(12):1746. doi: 10.3390/plants14121746 (PMC12196304; doi:10.3390/plants14121746)
Supplement: Supplementary file 1 [file plants-14-01746-s001.zip › plants-3653981-supplementary.pdf]

Supplementary Material

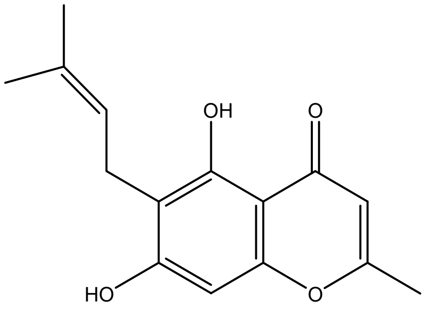

Peucenin **30**

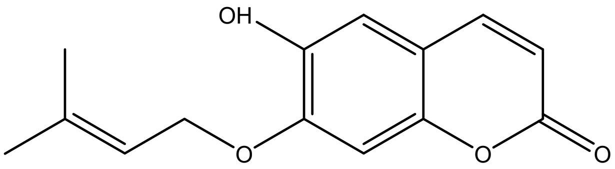

Prenyletin **31**

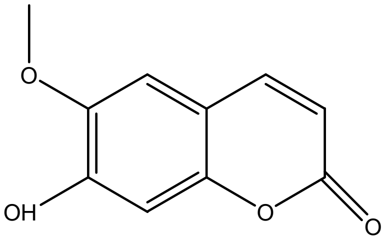

Scopoletin **32**

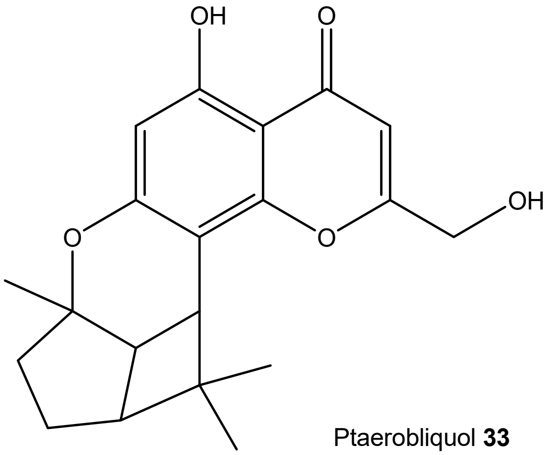

Ptaerobliquol **33**

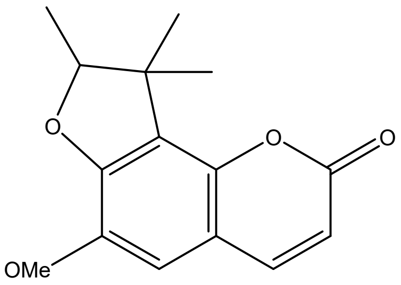

Nieshoutin or Cyclo-obliquetin **34**

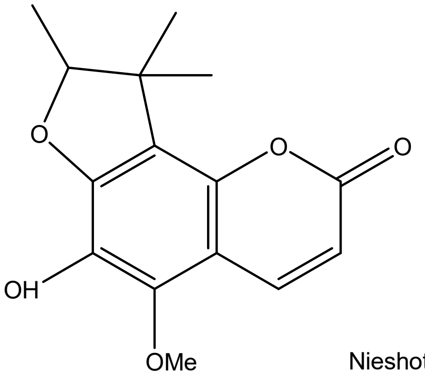

Nieshotol **35**

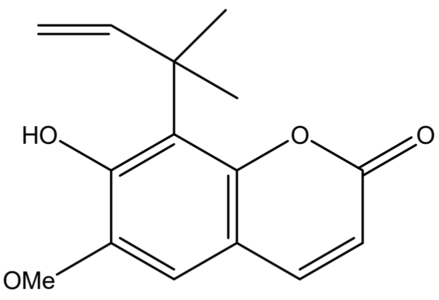

Obliquetin **36**

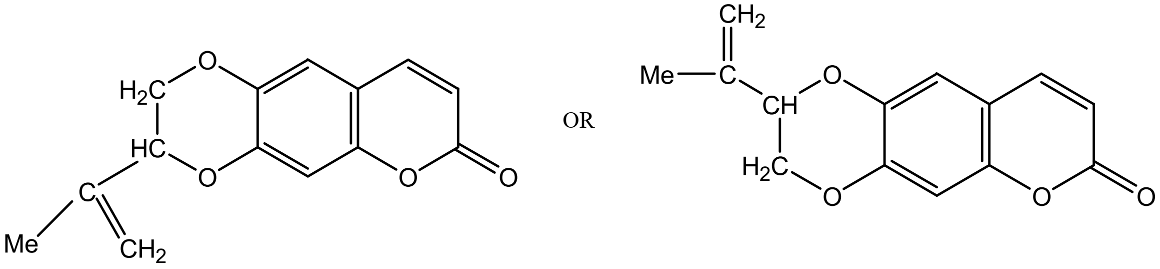

Obliquin **38**

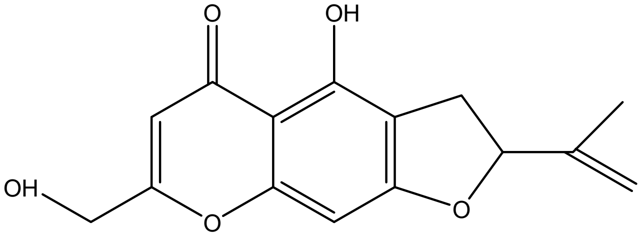

Umtatin **39**

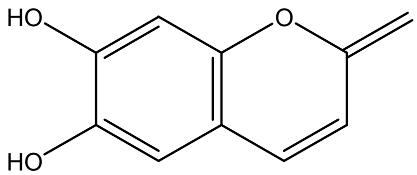

Aesculetin **37**

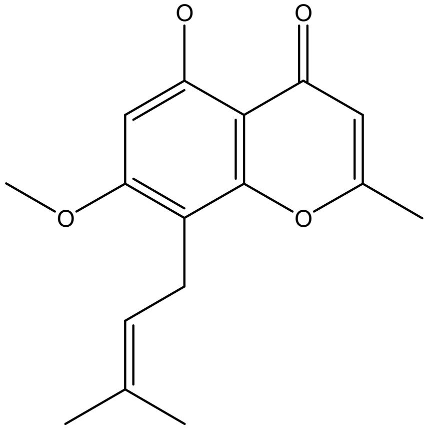

Heteropeucenin **41**

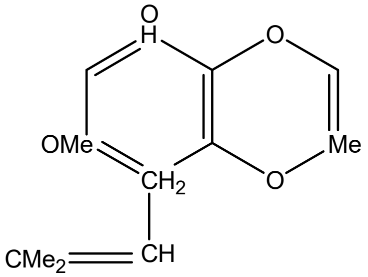

Heteropeucenin 7-methyl ether **40**

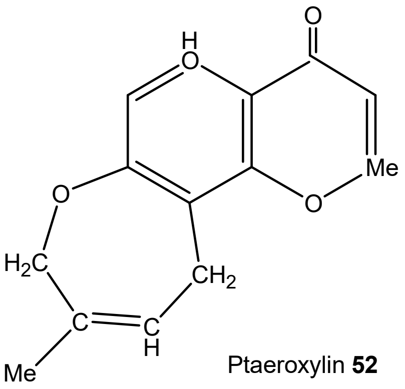

Ptaeroxylin **52**

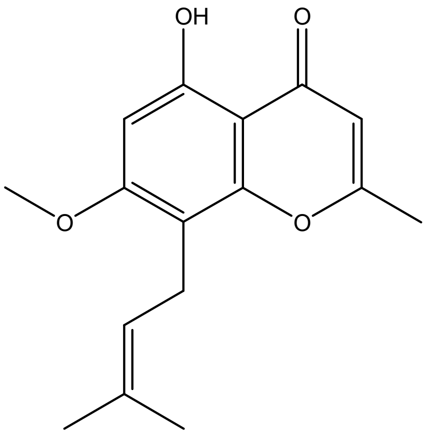

Heteropeucenin, methyl ether **42**

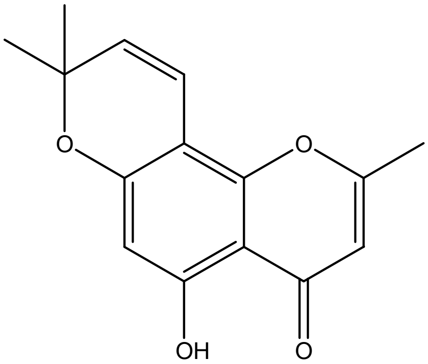

Alloptaeroxylin **43**

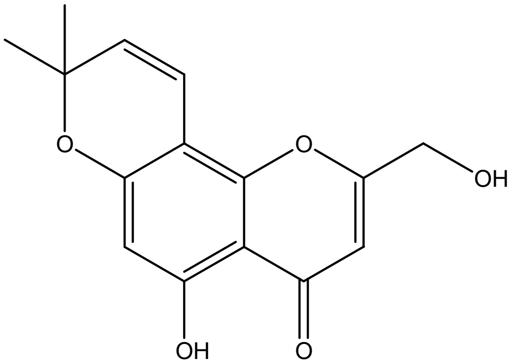

Ptaerochromenol **44**

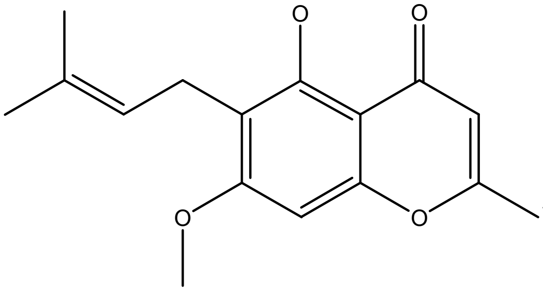

Peucenin, 7-methyl ether **45**

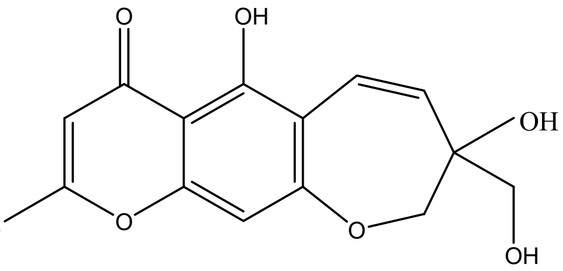

Ptaeroglycol **47**

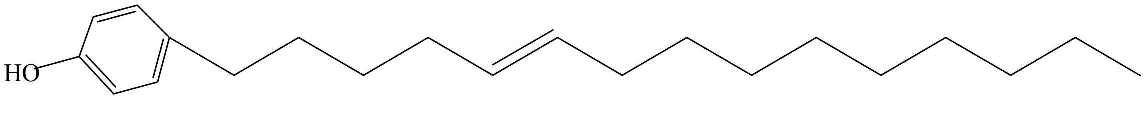

Obliquol **50**

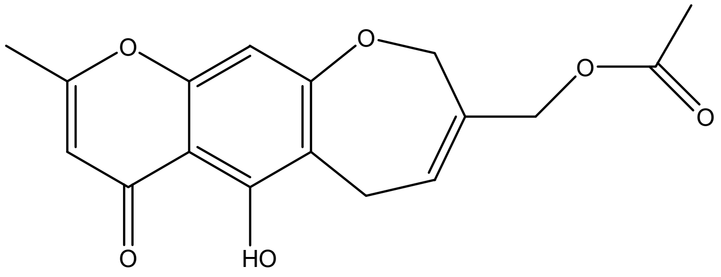

Obliquumol **53**

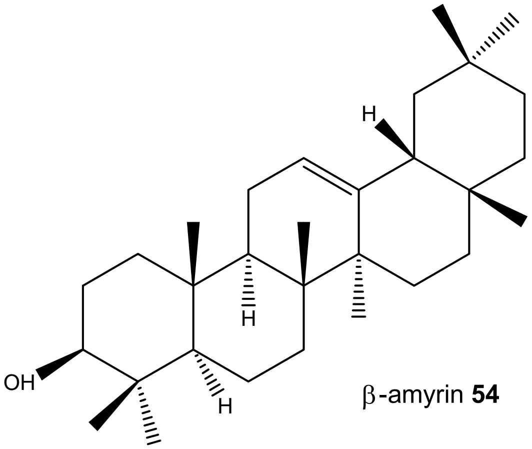

β-amyrin **54**

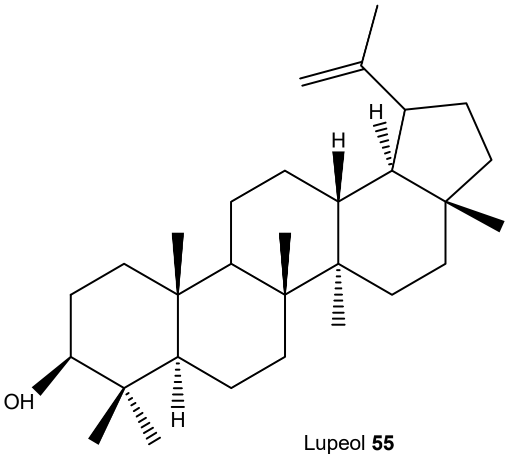

Lupeol **55**

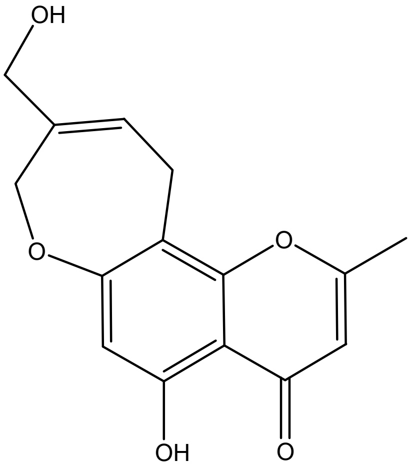

Eranthin **56**

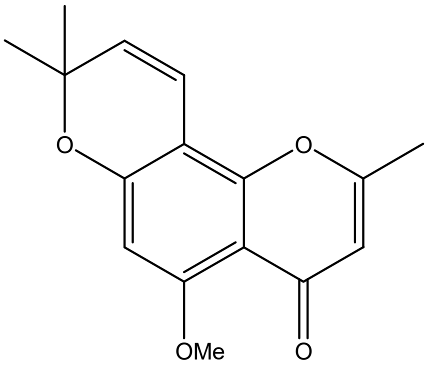

O-Methylalloptaeroxylin **57**

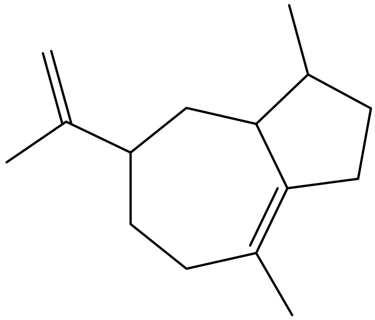

Guaia-1(10),11-diene **58**

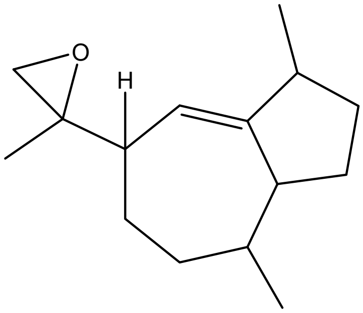

Gamma-Gurjunenepoxide-(2) **59**

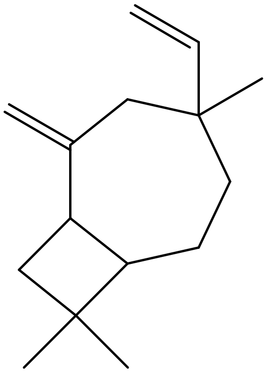

Bicyclo[5.2.0]nonane, 2-methylene-4,8,8-trimethyl-4-vinyl- **60**

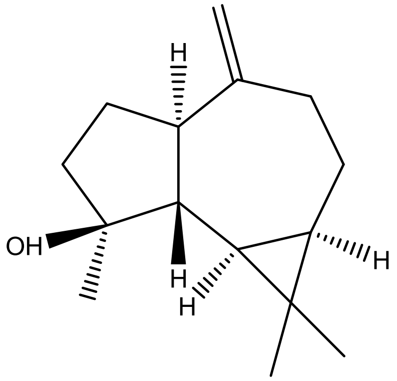

Spathulenol **61**

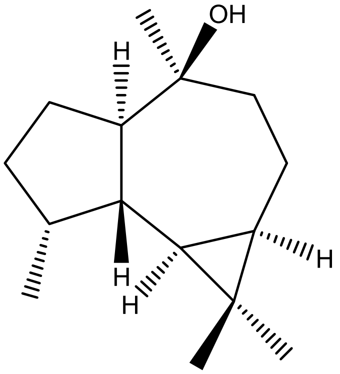

Epiglobulol **62**

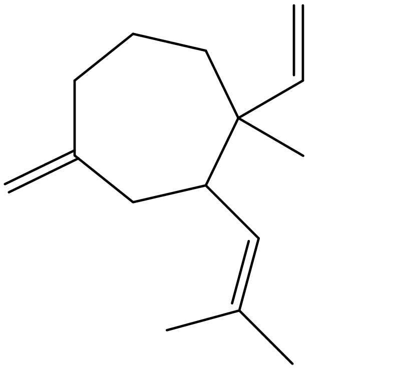

Cycloheptane, 4-methylene-1-methyl-2-(2-methyl-1-propen-1-yl)-1-vinyl- **63**

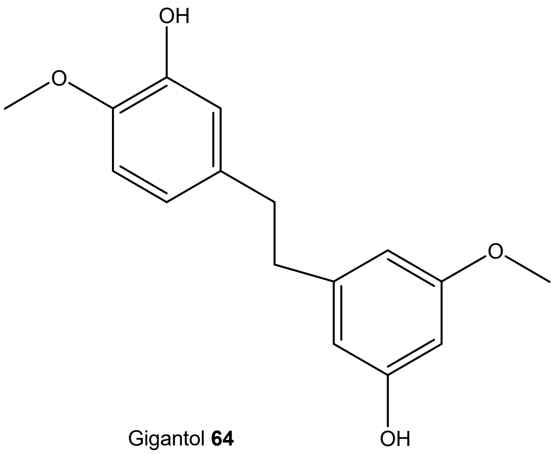

Gigantol **64**

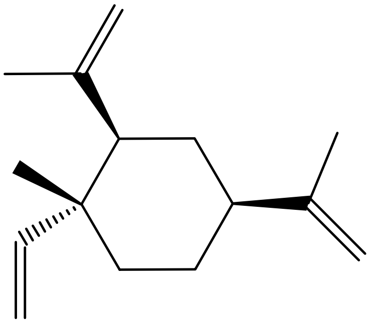

Cyclohexane, 1-ethenyl-1-methyl-2,4-bis(1-methylethenyl)-, [1S-(1α,2β,4β)]- **65**

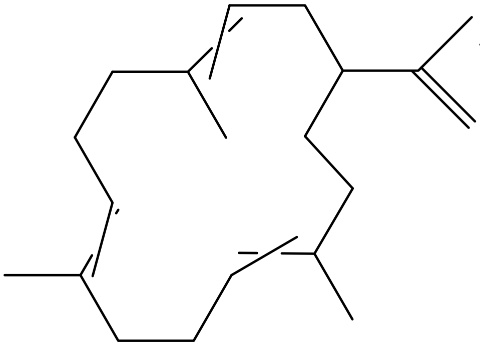

1,5,9-Cyclotetradecatriene, 1,5,9-trimethyl-12-(1-methylethenyl)- **66**

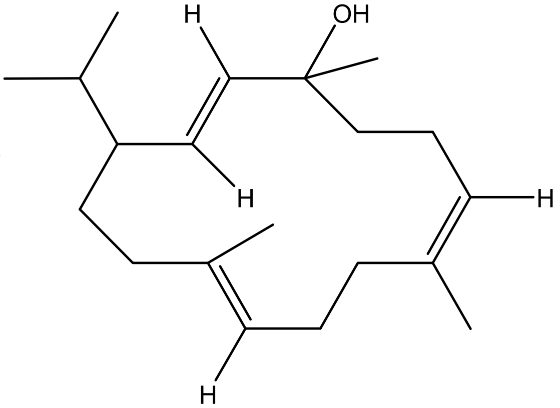

Thunbergol **67**

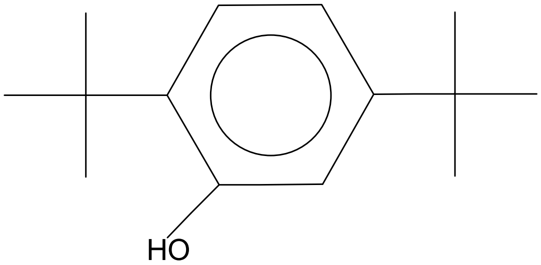

Phenol, 2,5-bis(1,1-dimethylethyl)- **75**

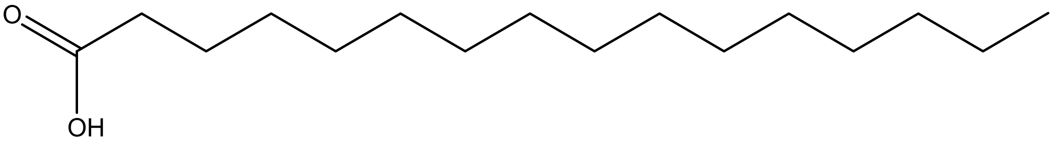

n-Hexadecanoic acid **68**

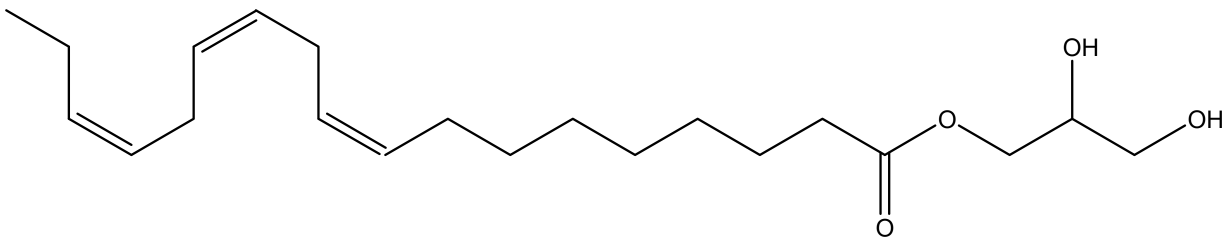

9,12,15-Octadecatrienoic acid,2,3-dihydroxypropyl ester, (Z,Z,Z)- **69**

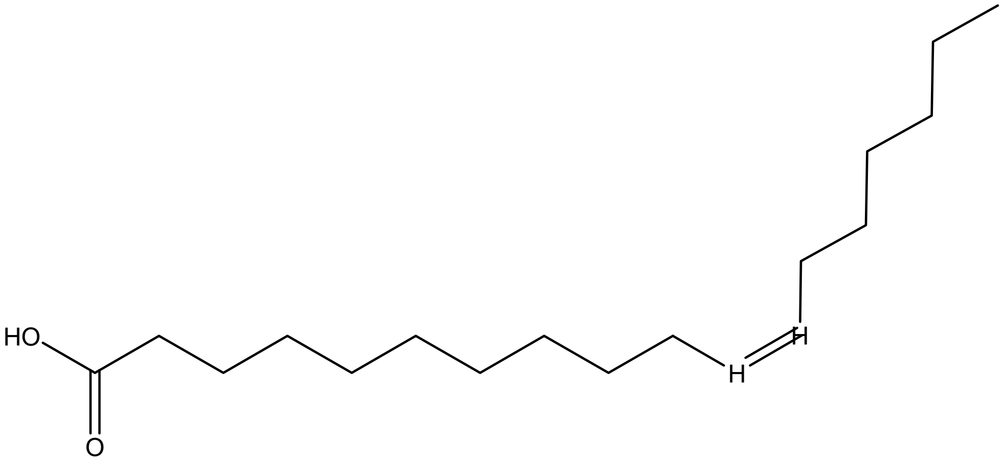

Vaccenic acid, *cis*- **70**

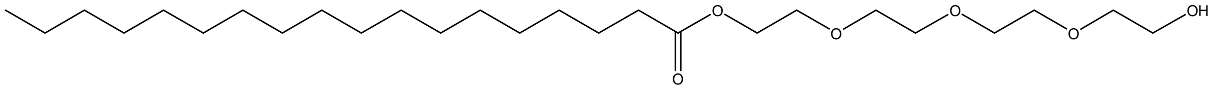

Octadecanoic acid,2-[2-[2-(2-hydroxyethoxy)ethoxy]ethoxy]ethyl ester **71**

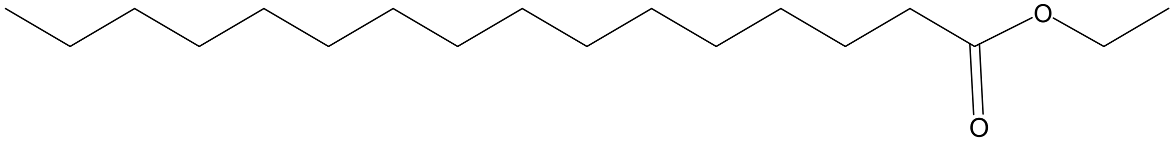

Hexadecanoic acid, ethyl ester **72**

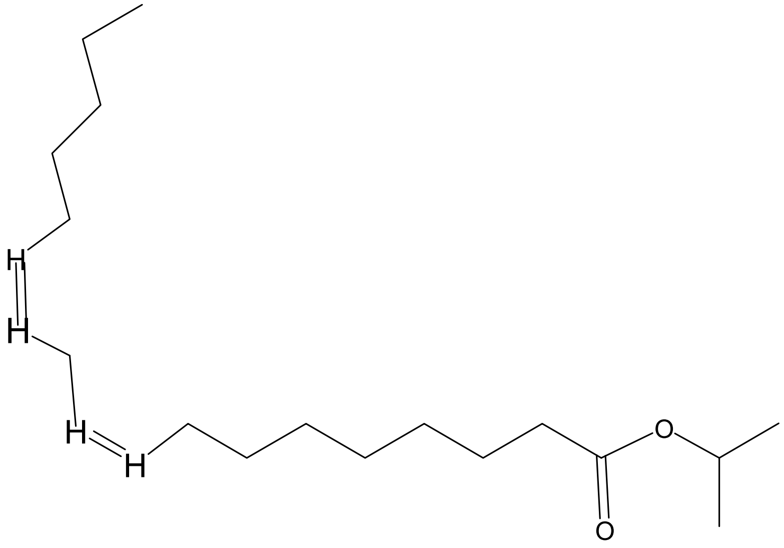

Isopropyl Linoleate **73**

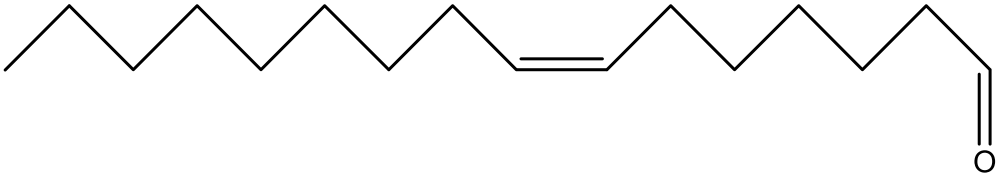

7-Hexadecenal, (Z)- **74**

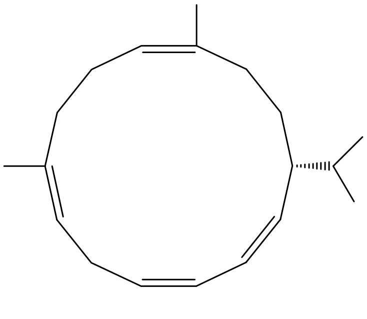

1,3,6,10-Cyclotetradecatetraene,
3,7,11-trimethyl-14-(1-methylethyl)- **76**

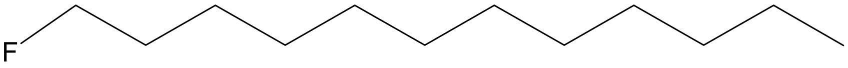

Dodecane,1-fluoro- **77**

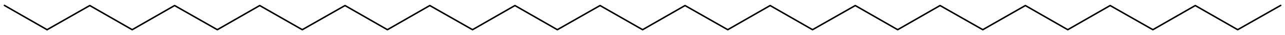

Hentriacontane **78**

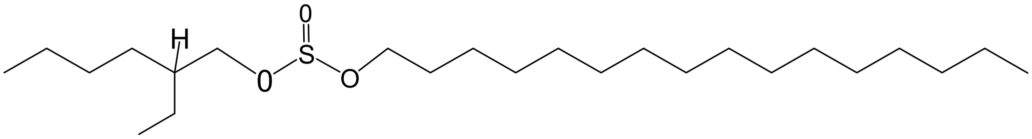

Sulfurous acid, 2-ethylhexyl hexadecyl ester **79**

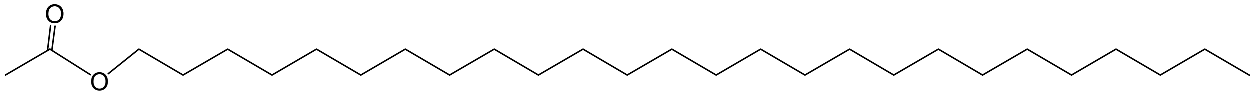

Hexacosyl acetate **80**

Figure S1. Structures of the isolated or tentatively identified compounds from *Ptaeroxylon obliquum*.
